# Striatum-related spontaneous coactivation patterns predict treatment response on positive symptoms of drug-naive first-episode schizophrenia with risperidone monotherapy

**DOI:** 10.3389/fpsyt.2023.1093030

**Published:** 2023-03-15

**Authors:** Xiaofen Zong, Kai Wu, Lei Li, Jiangbo Zhang, Simeng Ma, Lijun Kang, Nan Zhang, Luxian Lv, Deen Sang, Shenhong Weng, Huafu Chen, Junjie Zheng, Maolin Hu

**Affiliations:** ^1^Department of Psychiatry, Renmin Hospital of Wuhan University, Wuhan, China; ^2^High-Field Magnetic Resonance Brain Imaging Key Laboratory of Sichuan Province, School of Life Sciences and Technology, University of Electronic Science and Technology of China, Chengdu, China; ^3^Department of Psychiatry, Henan Mental Hospital, The Second Affiliated Hospital of Xinxiang Medical University, Xinxiang, China; ^4^Early Intervention Unit, Department of Psychiatry, Affiliated Nanjing Brain Hospital, Nanjing Medical University, Nanjing, China; ^5^Functional Brain Imaging Institute of Nanjing Medical University, Nanjing, China

**Keywords:** schizophrenia, striatum, resting-state network, network dynamics, coactivation patterns, risperidone

## Abstract

**Background:**

Evidence from functional magnetic resonance imaging (fMRI) studies of schizophrenia suggests that interindividual variation in the stationary striatal functional circuit may be correlated with antipsychotic treatment response. However, little is known about the role of the dynamic striatum-related network in predicting patients’ clinical improvement. The spontaneous coactivation pattern (CAP) technique has recently been found to be important for elucidating the non-stationary nature of functional brain networks.

**Methods:**

Forty-two drug-naive first-episode schizophrenia patients underwent fMRI and T1W imaging before and after 8 weeks of risperidone monotherapy. The striatum was divided into 3 subregions, including the putamen, pallidum, and caudate. Spontaneous CAPs and CAP states were utilized to measure the dynamic characteristics of brain networks. We used DPARSF and Dynamic Brain Connectome software to analyze each subregion-related CAP and CAP state for each group and then compared the between-group differences in the neural network biomarkers. We used Pearson’s correlation analysis to determine the associations between the neuroimaging measurements with between-group differences and the improvement in patients’ psychopathological symptoms.

**Results:**

In the putamen-related CAPs, patients showed significantly increased intensity in the bilateral thalamus, bilateral supplementary motor areas, bilateral medial, and paracingulate gyrus, left paracentral lobule, left medial superior frontal gyrus, and left anterior cingulate gyrus compared with healthy controls. After treatment, thalamic signals in the putamen-related CAP 1 showed a significant increase, while the signals of the medial and paracingulate gyrus in the putamen-related CAP 3 revealed a significant decrease. The increase in thalamic signal intensity in the putamen-related CAP 1 was significantly and positively correlated with the percentage reduction in PANSS_P.

**Conclusion:**

This study is the first to combine striatal CAPs and fMRI to explore treatment response-related biomarkers in the early phase of schizophrenia. Our findings suggest that dynamic changes in CAP states in the putamen-thalamus circuit may be potential biomarkers for predicting patients’ variation in the short-term treatment response of positive symptoms.

## Introduction

Schizophrenia is an extremely debilitating psychiatric disorder that has a lifetime prevalence of approximately 0.5%. A considerable body of new evidence from functional magnetic resonance imaging (fMRI) studies supports that striatal functional connectivity (FC) is disrupted in this disease (1–3). Most antipsychotic agents, which compose first-line treatments for schizophrenia, essentially block dopamine D2 receptors in the striatum. It has been suggested that striatal dopamine is central to both schizophrenia pathology and antipsychotic action (1), and interindividual variation in striatal functional circuit is disrupted in schizophrenia and correlated with antipsychotic treatment response (4–6).

Pharmacoimaging studies based on fMRI data have preliminarily revealed antipsychotic agent treatment response-related alterations in FC within striatal subregions (4) as well as between striatal and extrastriatal regions such as the corticostriatal circuitry (7). The premise of these studies is that blood oxygenation level-dependent (BOLD) signals (8), which are obtained without explicit tasks or stimulation during fMRI scans, are temporally associated within functionally related brain areas. Despite a significant amount of existing research, the role of striatal FC as an antipsychotic treatment response biomarker is still being elucidated. The principal imaging approaches available for this goal have generally assumed stationarity that the temporal series do not alter their nature over time. The brain, however, is a complicated system characterized by dynamic FC (9). It has been found that interareal BOLD signal correlations demonstrated large variation over the timescale of tens of seconds to several minutes, confirming the non-stationary characteristic of FC (10), which provides an opportunity to increase the subtle information about the FC network computed during the analysis process.

Previous methods for depicting the non-stationary nature of FC have depended primarily on detecting temporal variability in BOLD signal coherences utilizing sliding window analysis or time–frequency analysis (10–13). These approaches are limited to a few regions of interest (ROIs) and their pairwise relationship. Another shortcoming of these time-domain approaches is the potential confounding effects of non-neurogenic signals such as instrumental drift, head motion, and thermal noise, which were found to be difficult to distinguish from the signals of interest, especially when utilizing shorter sliding windows (10). Recently, the spontaneous coactivation pattern (CAP) technique (14–16) has played a vital role in elucidating the dynamic nature of non-stationary resting state brain networks. As a data-driven approach, the CAP technique, which depends on few mathematical presumptions, is free of the confounding effect from the length of the sliding time window. Therefore, CAP analysis has been increasingly utilized to investigate aberrant brain network dynamics in schizophrenia (15) and other neuropsychiatric disorders such as depression (17), autism (18, 19), and Alzheimer’s disease (20). Yang et al. (15) found that patients with schizophrenia spend more time in CAP states involving the default mode and salience networks, but less time in CAP states involving the fronto-parietal network, and the abnormal dynamic characteristics of functional CAPs are correlated with patients’ symptom severity. However, to date, there is no direct evidence for the role of dynamic striatal FC in predicting antipsychotic treatment response.

This study therefore integrated striatal CAP measures to investigate treatment response- and schizophrenia-related biomarkers in the early phase of schizophrenia. All the patients in this study were treated with risperidone monotherapy for 8 weeks to control for the confounding effects of multidrug treatment on treatment response. Given the previous evidence (1, 4–6), we tested the hypothesis that striatal CAP abnormalities would be pathological traits characterizing schizophrenia and would relate to individual-level variation in antipsychotic response. To test the hypotheses, we applied the robust analytical approach CAPs (14, 15) and fMRI modalities to infer the aberrant state dynamics of the striatum. Given that the striatum consists of the neostriatum (caudate and putamen) and paleostriatum (pallidum) (21), we extracted the 3 regions of interest from the Automated Anatomical Labeling (AAL) template and analyzed their CAPs and dynamic states. The overlapping brain regions, of which activity in striatal CAPs showed significant alterations both at baseline and after treatment, were considered to be involved in both pathological and pharmacological mechanisms, and viewed as effective therapeutic targets. Then, Pearson’s correlation analysis was applied to compute the associations between neuroimaging measurements (including CAPs and dynamic CAP states) of the overlapping regions and the improvement of patients’ psychopathological symptoms to define the treatment response biomarkers.

## Materials and methods

### Participants

We recruited 42 treatment-naive patients with first-episode schizophrenia (Patients-0W) and 38 education-, gender-, and age- matched healthy controls in the Second Affiliated Hospital of Xinxiang Medical University in China from 2012.12 to 2014.01, China. This fMRI dataset has been used previously by our group (22, 23). Experienced psychiatrists diagnosed patients according to the Structured Clinical Interview for DSM-IV-TR. The illness duration of all the patients in this study was less than 12 months. SCID-non-patient edition was used to scan controls to confirm that they had no history of other psychiatric or neurological disorders. In this observational cohort study, patients were treated with risperidone, and then followed for 8 weeks.

The study was approved by the Ethics Committee of Henan Mental Hospital and Second Xiangya Hospital of Central South University (protocol code S088, 2012). It was conducted in accordance with the Declaration of Helsinki. Written informed consent was acquired from all the participants involved in the study.

### Therapy and clinical assessments

The initial dose of risperidone was 2 or 1 mg per day within the first week. After 1 week, a slow titration was then utilized, and the risperidone dose was increased at 1-week intervals until patients showed satisfactory improvement. After 4 weeks, patients who did not exhibit obvious improvement were prescribed up to 6 mg/day of risperidone. All patients were treated with risperidone monotherapy for 8 weeks. Patients were also treated with benzhexol if they had extrapyramidal symptoms. During the 8-week intervention, some patients (*n* = 19) were given at least one dose (ranging from 1 to 2 mg) of lorazepam; however, this medication could not be taken within 1 week before the second scan. Patients were also not allowed to use antidepressants or mood stabilizers during the intervention period, as these medications are potential synergists. Treatment safety was evaluated weekly through clinical interviews with doctors.

Symptom severity was assessed both before and after 8 weeks of treatment on the day of MRI scanning by using the Positive and Negative Syndrome Scale (PANSS) (24), which primarily evaluates positive (PANSS-P, Items P1–P7) and negative (PANSS-N, Items N1–N7) symptom dimensions.

### Imaging data acquisition

The participants underwent T1 weighted (T1W) imaging and fMRI on a 3.0T Siemens MRI scanner (Verio) at the Magnetic Imaging Centre of the Henan Mental Hospital. A standard 16-channel head coil was utilized. We scanned patients both at baseline and at the 8-week follow-up, while healthy controls only underwent the baseline scans. T1W images were obtained sagittally with a gradient echo pulse sequence. The detailed parameters were echo time (TE) = 2.52 ms, repetition time (TR) = 1,900 ms, field of view (FOV) = 250 mm × 250 mm, flip angle (FA) = 9°, slice gap = 0 mm, slice thickness (ST) = 1.0 mm, and number of slices = 176. Resting-state fMRI data were acquired with a gradient-echo echo-planar imaging sequence. The parameters were TE = 30 ms, TR = 2,000 ms, FOV = 240 mm × 240 mm, FA = 90°, time point = 240, matrix size = 64 × 64, ST = 3 mm, voxel size = 3.75 mm × 3.75 mm × 3 mm, and slice gap = 0. Patients were required to relax and keep their eyes closed while staying awake during the scanning.

Four patients withdrew from the follow-up scans. We therefore obtained imaging data from 42 patients at baseline (patients 0W) and 38 patients at the 8-week follow-up (patients-8W) as well as 38 healthy volunteers.

### Image data preprocessing

We used the Data Processing Assistant for Resting-State fMRI (DPARSF,^1^ DPARSF5.2) (25) to preprocess the fMRI images. The DPARSF is a toolbox for MATLAB (R2019b) and Data Processing and Analysis of Brain Imaging (DPABI)^2^ (26). Slice timing and EPI undistortion correction were performed. Any subject with head motion >1.5° rotation or >1.5 mm translation in any direction was removed. We regressed out the motion parameters, white matter, and CSF from the time series. All subjects’ functional images were normalized to the Montreal Neurological Institute (MNI) space with the standard EPI template (resampled voxel size 3 × 3 × 3 mm^3^). We also conducted T1 coregistration analysis to preprocess the imaging data. The resampled images were then smoothed with the Gaussian kernel and detrended to remove the linear drift of BOLD signals.

The baseline fMRI data of four patients failed to meet the standards of quality control, and these four patients withdrew from the follow-up imaging scans.

### Extraction of ROI time series

We imported the AAL template and extracted the time series of the 3 ROIs (putamen, pallidum, and caudate) in DPABI. The MNI coordinates and the position of the 3 ROIs in the Automated Anatomical Labeling (AAL) template are shown in Supplementary Figure 1. DPABI was used to extract the time series of the 3 ROIs and all other brain regions. We averaged the BOLD signal intensity of all the time points (fMRI time frames) of each ROI.

### Extraction and normalization of CAP maps

With the goal of extracting striatum-related spontaneous CAPs, we first chose the top 15% of time points based on previous evidence (14), which demonstrated that the similarity (spatial associations) between the ROI-seeded association map utilizing all time points and the averaged signals of the selected time points rapidly increased while including more time points by decreasing the selection threshold; the spatial associations then reached a plateau after including ∼ 15% of all time points. Then, we selected the top 36 (240 × 15% = 36) time points for the 3 ROIs as the highly activated state. The similarity between our approach and a previous study (14) is the setting of the threshold (top 15% time points), while the difference lies in the selection of the seed-regions.

The three-dimensional brain maps of the 36 highly activated time points from the whole-brain voxel level were extracted and normalized by subtracting the mean BOLD signal intensity at each time point and dividing that value by the standard deviation. We averaged the normalized BOLD signal intensity of the highly activated time points on a regional level. The 3 ROI-related spontaneous CAPs in the control group are shown as an example in Supplementary Figure 2.

We compared the differences in striatum-related CAPs between Patients-0W and controls as well as between Patients-0W and Patients-8 by performing independent samples *t*-tests and paired *t*-tests, respectively. GRF correction was performed as well, with voxel level *P* < 0.01, cluster level *P* < 0.05, and voxel number >50.

### Cluster analysis of dynamic CAP states

To extract further information about the dynamic variations in longitudinal ROI-related CAPs after treatment, after extracting striatum-related spontaneous CAPs, we then classified the 36 detected highly activated time points (in the ROI-related CAPs) according to their spatial similarity utilizing the k-means clustering approach. The above approach, i.e., clustering the top 15% of time points, has been applied previously (14). This process was performed on Dynamic Brain Connectome (Dynamic BC),^3^ a MATLAB toolbox (Supplementary Figure 3). The CAPs of both Patients-0W and Patients-8W were classified into three states. The brain maps in each state (CAP 1, CAP 2, and CAP 3) were extracted and averaged for Patients-0W and Patients-8W. We used paired sample *t*-tests to compare the longitudinal changes in BOLD signal intensity in each state, with GRF correction, voxel level *P* < 0.01, cluster level *P* < 0.05, and voxel number >50.

### Investigating treatment response biomarkers

Overlapping areas between regions with case-control differences and those with longitudinal differences were viewed as effective treatment targets. We used Pearson’s correlation analysis to compute the correlations between patients’ clinical symptom improvement and longitudinal changes in imaging markers of the overlapping regions, including longitudinal alterations in CAPs and dynamic CAP states.

## Results

### Demographics and clinical symptoms

We detected no significant differences in the between-group comparisons of age, gender, handedness, and education data of patients and controls (*Ps* > 0.05; Supplementary Table 1). Patients showed significant clinical improvement in positive symptoms (*Ps* < 0.001; Supplementary Table 2), while their negative symptoms showed no significant alterations (*P* > 0.05; Supplementary Table 2).

### Abnormal striatum-related spontaneous CAPs in schizophrenia

**FIGURE 1:**
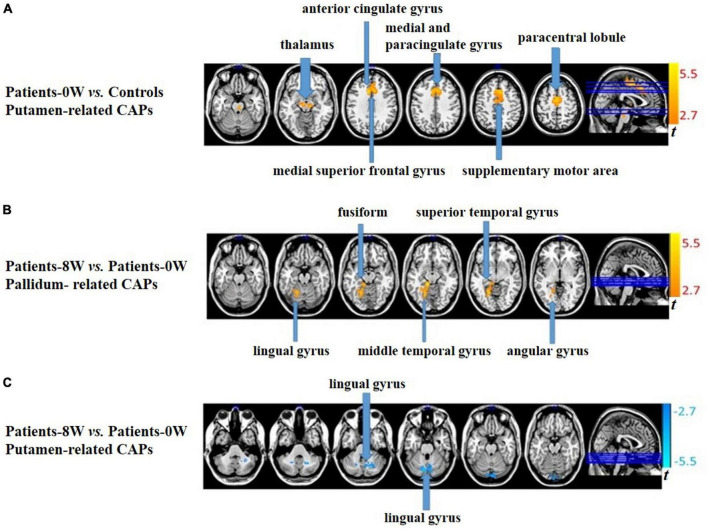
Between-group comparisons of striatum-related spontaneous coactivation patterns (CAPs). **(A)** Patients showed significantly increased signal intensity in the putamen-related CAPs at baseline. **(B)** Patients had significantly increased signal intensity in the pallidum-related CAPs after treatment. **(C)** Patients had significantly decreased signal intensity in the putamen-related CAPs after treatment.

In the putamen-related CAPs, patients showed significantly increased intensity in the bilateral thalamus, bilateral supplementary motor area (SMA), bilateral medial, and paracingulate gyrus, left paracentral lobule, left medial superior frontal gyrus, and left anterior cingulate gyrus compared with healthy controls (GRF correction, voxel level *P* < 0.01, cluster level *P* < 0.05, and voxel number >50, Figure 1A and Supplementary Table 3). There were no significant between-group differences in the caudate- or pallidum-related CAPs.

### CAP alterations after treatment and associations with treatment response

In pallidum-related CAPs, patients had significantly increased intensity in the left lingual, fusiform, middle temporal, superior temporal, and angular gyrus after treatment (GRF correction, voxel level *P* < 0.01, cluster level *P* < 0.05, and voxel number >50, Figure 1B and Supplementary Table 4). In the putamen-related CAPs, patients had significantly decreased intensity in the left lingual gyrus (GRF correction, voxel level *P* < 0.01, cluster level *P* < 0.05, and voxel number >50, Figure 1C and Supplementary Table 4). Regarding the caudate-related CAPs, patients showed no significant longitudinal alterations.

There were no overlapping regions between areas with case-control differences and those with 8-week longitudinal differences in patients. We therefore did not further analyze the correlations between patients’ clinical symptom improvement and the longitudinal changes in neuroimaging measurements.

### Longitudinal alterations of striatum-related CAP states and associations with treatment response

We detected significant longitudinal differences in the three subregion-related dynamic CAP states (GRF correction, voxel level *P* < 0.01, cluster level *P* < 0.05, and voxel number >50), except the putamen-related CAP 2 (see Table 1).

**TABLE 1:** Longitudinal alterations of the three subregions-related CAP states.

ROIs	State	Brain regions with significance	Hemisphere	MNI coordinates			*t* ^a^
				***X***	***Y***	***Z***	
Caudate patient-8w vs. patient-0w	1	Pars triangularis	Right	60	27	6	−6.92
		Post-central gyrus	Left	−21	−27	69	−8.59
	2	Middle occipital gyrus	Left	−18	−96	9	8.58
			Right	27	−87	18	7.06
		Fusiform gyrus	Right	27	−84	−3	8.25
		Superior occipital gyrus	Right	24	−90	15	7.97
		Cuneus	Right	6	−90	−24	8.1
		Lingual gyrus	Right	12	−75	−3	9.91
	3	Inferior occipital gyrus	Left	−42	−72	−6	6.32
		Supplementary motor area	Left	−3	12	57	7.23
Pallidum patient-8w vs. patient-0w	1	Lingual gurus	Left	−12	−48	−6	8.16
			Right	21	−51	−6	10.78
		Fusiform	Left	−21	−54	−15	10.24
			Right	30	−63	−15	8.94
		Middle occipital gyrus	Left	−27	−84	33	6.67
		Superior parietal gyrus	Left	−30	−63	66	−6.96
		Middle temporal gyrus	Right	57	−36	−9	−8.16
	2	Fusiform	Left	−24	−72	−12	6.16
	3	Fusiform	Left	−27	−69	−12	6.96
			Right	27	−45	−12	6.67
Putamen patient-8w vs. patient-0w	1	Insular	Left	−27	18	3	9.20
		Thalamus^b^	Left	−12	−9	3	5.92
		Pars triangularis	Left	−42	42	9	7.26
		Precuneus	Left	−6	−51	63	−7.17
			Right	6	−48	75	−7.32
		Post-central gyrus	Right	21	−39	66	−5.85
	3	Insular	Left	−42	15	3	−11.32
			Right	39	6	0	−10.95
		Medial and paracingulate^b^	Left	0	9	36	−9.33
			Right	9	15	36	−10.10

CAPs, coactivation patterns; ROI, regions of interest; MNI, Montreal Neurological Institute. ^a^GRF correction, voxel level *P* < 0.01, cluster level *P* < 0.05, and voxel number >50. ^b^Thalamus, and medial and paracingulate gyrus are the overlapping brain regions between areas with case-control differences, and that with 8-week longitudinal differences in the patient group, which were viewed as effective therapeutic targets.

**FIGURE 2:**
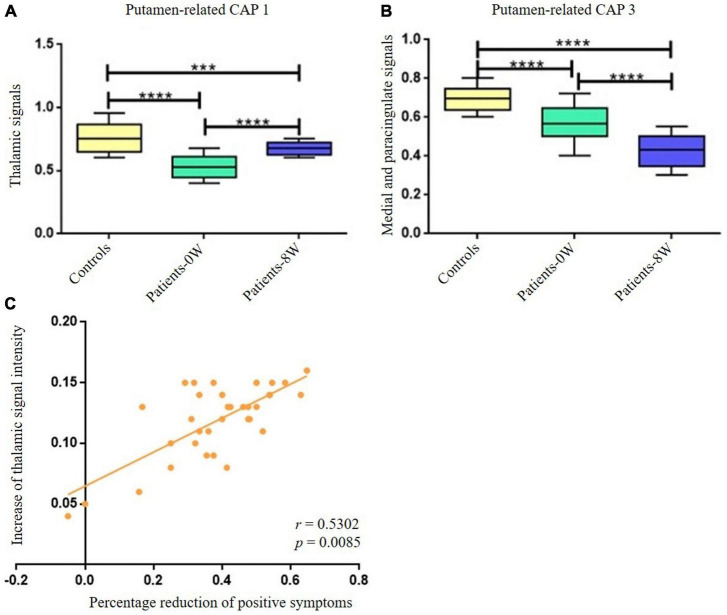
Longitudinal alterations of striatum-related CAP states and associations with treatment response. **(A)** Thalamic signal in the Putamen-related CAP 1 showed significant increase after treatment. **(B)** Medial and paracingulate gyrus signal in the putamen-related CAP 3 showed significant decrease after treatment. **(C)** The increase of thalamic signal intensity demonstrated significantly positive correlations with the percentage reduction of patients’ positive symptoms (*P* = 0.0170 with Bonferroni correction). ***Represents *P* < 0.001, ****represents *P* < 0.0005.

By comparing the differential brain regions of Patients-0W vs. Controls (Figure 2A) and that of Patients-0W vs. Patients-8W (Table 1), we found that thalamus (detected in the putamen-related CAP 1, Table 1) and medial and paracingulate gyrus (detected in the putamen-related CAP 3, Table 1) were the overlapping brain regions. Thalamic signals in the putamen-related CAP 1 showed a significant increase (GRF correction, voxel level *P* < 0.01, cluster level *P* < 0.05, and voxel number >50, Figure 2A), while the signals of the medial and paracingulate gyrus in the putamen-related CAP 3 significantly decreased after treatment (GRF correction, voxel level *P* < 0.01, cluster level *P* < 0.05, and voxel number >50, Figure 2B).

The increase in thalamic signal intensity in the putamen-related CAP 1 showed significantly positive correlations with the percentage reduction (Patients-0W–Patients–8W/Patients-0W) of PANSS_P (*r* = 0.5302, *P* = 0.017 with Bonferroni correction, Figure 2C). We did not detect significant relations between the percentage reduction of PANSS_P and the signal intensity decrease of the medial and paracingulate gyrus in the putamen-related CAP 3 (*P* > 0.05 with Bonferroni correction).

## Discussion

This study first integrates striatal CAP measures and fMRI modalities to explore treatment response biomarkers in the early phase of schizophrenia. As hypothesized, patients in the early phase of schizophrenia had striatal CAP abnormalities, parts of which were correlated with their variation in antipsychotic response of positive symptoms.

The diverse pathways and functional contributions of the striatum, coupled with known dopaminergic function differences in schizophrenia patients (1, 4), imply that the striatum may be a vital contributor to the etiology of this disease. Consistently, our current study identified that patients had increased signal intensity in the putamen-related CAPs, which involved the bilateral thalamus, SMA, medial and paracingulate gyrus, as well as the left paracentral lobule, medial superior frontal, and anterior cingulate gyrus. These findings emphasized the abnormal CAPs in the striatal-thalamic-cortico loop in individuals in the early phase of schizophrenia. This circuit plays an important role in modulating sustained attention, and functional and anatomical abnormalities in this circuitry have been confirmed in extensive MRI studies of schizophrenia (4, 27–30). We utilized a novel striatal CAP neuroimaging biomarker to demonstrate functional abnormalities associated with the pathologic mechanism of schizophrenia in the known striatal-thalamic-cortico circuit. In brief, our current study first identified schizophrenia pathology as resulting from striatal CAPs.

Our current study demonstrated that putamen-related thalamus signals were significantly increased after treatment, and the alterations were associated with patients’ positive symptom improvement. Notably, it is possible that aberrant activity of dopamine in mesolimbic neural circuits may be correlated with psychotic symptoms of schizophrenia (1, 31), and antipsychotic treatment may alter the mesolimbic neural pathways and thus improve patients’ psychotic symptoms. The striatum is an important mesolimbic structure. More importantly, the putamen, part of the dorsal striatum, is the gateway to basal nuclei (32); it receives excitatory afferents from the thalamus and cortical regions and forms the origin of the indirect and direct circuits (33–35). Our findings of the associations between the increase in putamen-related thalamic signal intensity and patients’ positive symptom alterations support the hypothesis that dynamic striatal CAP abnormalities are related to individual-level variation in antipsychotic response.

Approximately 1/3 of schizophrenia patients are defined as non-responders to first-line antipsychotic medications (36). Therefore, there is an urgent need for potential biomarkers to guide individualized precision therapy. In this regard, dynamic changes in CAP states in the putamen-thalamus circuit, which unveiled significant associations with antipsychotic response in the positive symptom dimension, could hold promise as biomarkers for guiding treatment choices. However, these results only apply to acute phase antipsychotic treatment. It remains unclear whether meaningful striatal CAP alterations in the brain can predict patients’ long-term treatment outcomes.

The findings in this study should be interpreted in light of several limitations. First, the sample size is small due to the challenges of recruiting drug-naive first-episode schizophrenia patients who would be treated with antipsychotic monotherapy. Future studies recruiting large samples are warranted. Second, the healthy volunteers were not scanned two times. Thus, the detected longitudinal striatal CAP differences in the patient group should be interpreted with caution, as it cannot be excluded that the differences are induced by the test-retest effect and longitudinal time alterations. Actually, it would be better to include patient control groups taking placebo, which could exclude (to a great extent) other causes of longitudinal alterations in imaging indices, including both test-retest effects, and progressive course of the illness (even over only 8 weeks). However, such a study design is clinically unethical given that known effective antipsychotic agents cannot be withheld from individuals with schizophrenia for 8 weeks. Third, there were no other patient control groups receiving other types of antipsychotic agents. We therefore cannot clarify whether the effects detected in our current study are specific to risperidone. Fourth, participants in this study might have been acclimated to the MRI scanning environment during the second scan, which may also influence our findings. Fifth, while our findings are consistent with a growing body of literature, replication in other exploratory cohorts is needed.

In conclusion, this study is the first to combine striatal CAPs and fMRI to explore schizophrenia- and treatment response-related biomarkers in the early phase of schizophrenia. Our findings suggest that patients in the early phase of schizophrenia had striatal CAP abnormalities, and dynamic changes in CAPs in the putamen-thalamus circuit may be potential biomarkers predicting patients’ variation in the short-term treatment response of positive symptoms.

## Data availability statement

The raw data supporting the conclusions of this article will be made available by the authors, without undue reservation.

## Ethics statement

The studies involving human participants were reviewed and approved by the Ethics Committee of Henan Mental Hospital and the Second Xiangya Hospital of Central South University. The patients/participants provided their written informed consent to participate in this study.

## Author contributions

XZ, MH, HC, JJZ, and SW conceived and designed the study. KW, JBZ, SM, LK, NZ, LL, LXL, and DS performed the investigation and data analysis. XZ, JJZ, and MH contributed to the interpretation of the data. XZ, MH, and KW wrote the manuscript. All authors contributed to the article and approved the submitted version.

## Footnotes

1
http://www.rfmri.org/dparsf

2
http://rfmri.org/DPABI

3
http://restfmri.net/forum/DynamicBC
