# Supplementary material for: Striatum-related spontaneous coactivation patterns predict treatment response on positive symptoms of drug-naive first-episode schizophrenia with risperidone monotherapy
Source: Front Psychiatry. 2023 Mar 15;14:1093030. doi: 10.3389/fpsyt.2023.1093030 (PMC10050338; doi:10.3389/fpsyt.2023.1093030)
Supplement: Supplementary file 1 [file Data_Sheet_1.doc]

**Striatum-related spontaneous coactivation patterns predict treatment response on positive symptoms of drug-naive first-episode schizophrenia with risperidone monotherapy**

Xiaofen Zong^1^, Kai Wu^2^, Lei Li^2^, Jiangbo Zhang^2^, Simeng Ma^1^, Lijun Kang^1^, Nan Zhang^1^, Luxian Lv^3^, Deen Sang^3^, Shenhong Weng^1^*, Huafu Chen^2^*, Junjie Zheng^4,5^* and Maolin Hu^1^*

^1^ Department of Psychiatry, Renmin Hospital of Wuhan University, Wuhan, China,

^2^ High-Field Magnetic Resonance Brain Imaging Key Laboratory of Sichuan Province, School of Life Science and Technology, University of Electronic Science and Technology of China, Chengdu, China,

^3^ Department of Psychiatry, Henan Mental Hospital, the Second Affiliated Hospital of Xinxiang Medical University, Xinxiang, China,

^4^ Early Intervention Unit, Department of Psychiatry, Affiliated Nanjing Brain Hospital, Nanjing Medical University, Nanjing, China,

^5^ Functional Brain Imaging Institute of Nanjing Medical University, Nanjing, China,

^6^ Department of Psychiatry, the Second Xiangya Hospital, Central South University, Changsha, China

*Correspondence: Maolin Hu, [humaolin@whu.edu.cn](mailto:humaolin@whu.edu.cn); Shenhong Weng, [wengshenhong@whu.edu.cn](mailto:wengshenhong@whu.edu.cn); Junjie Zheng, zjj5270@163.com; Huafu Chen, [chenhf@uestc.edu.cn](mailto:chenhf@uestc.edu.cn)

**SUPPLEMENTARY METHODS**

**Extraction and Normalization of CAP Maps**

The central coordinates of the 3 ROIs (putamen, pallidum and caudate) in the standard MNI template are: caudate (x=±14, y=0, z =21), putamen (x = ±27, y = 0, z = 5), pallidum (x = ±18, y = 0, z = 1), see Figure S1.


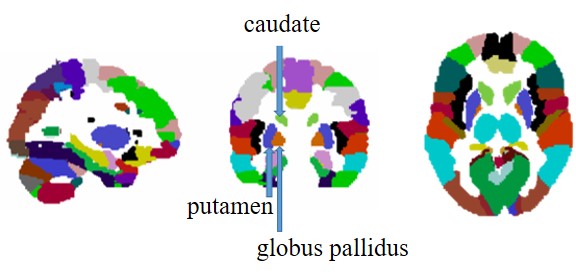


**Figure S1**. Location of three Striatal Subregions


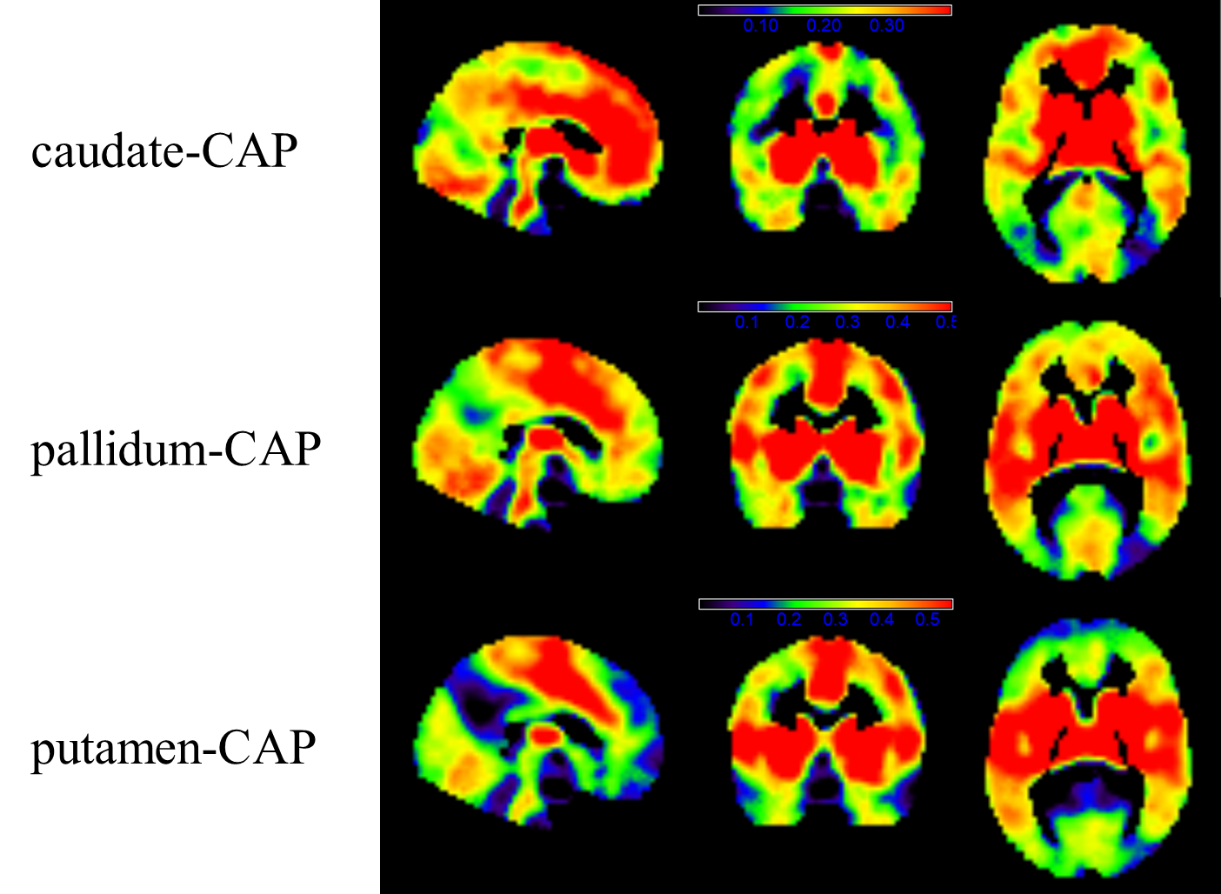


**Figure S2.** Striatal subregions-related spontaneous CAP maps in the control group

**Cluster Analysis of CAP states**

In order to further uncover the dynamic variations of treatment-related ROIs-CAPs, we classified the 36 highly activated time points (in the ROIs-related CAP maps) according to their spatial similarity utilizing the k-means clustering approach. This process was performed on Dynamic brain connectome (Dynamic BC, http://restfmri.net/forum/DynamicBC), a Matlab toolbox (Figure S3).
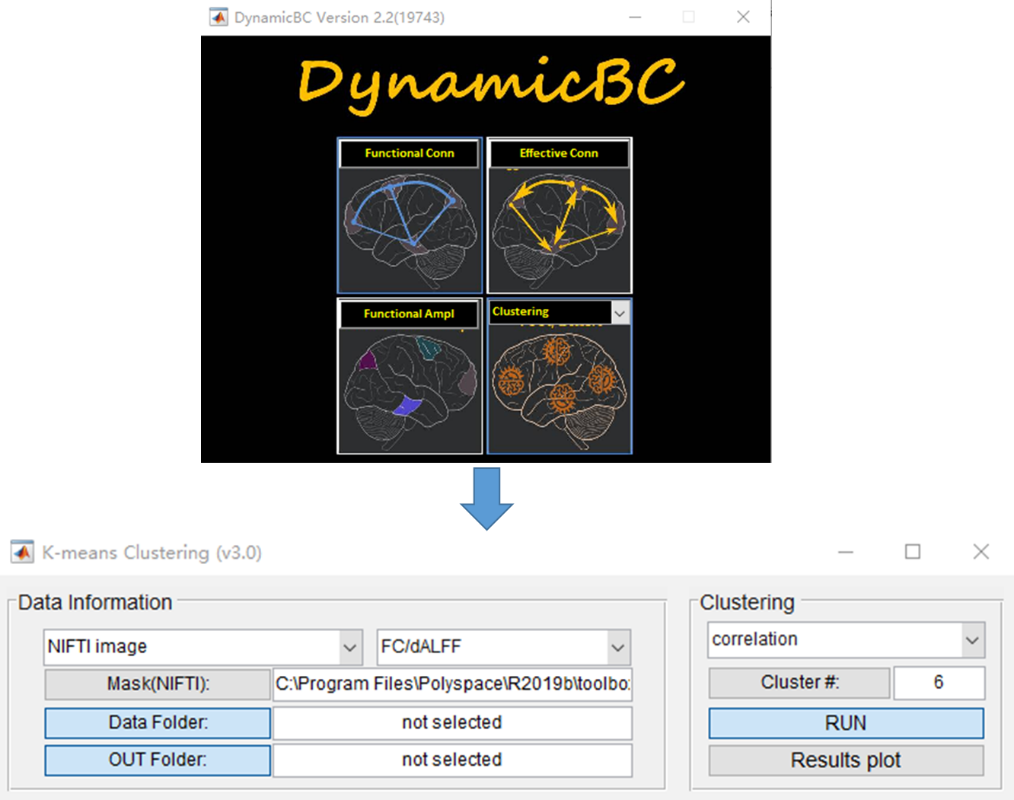


**Figure S3.** Cluster analysis of CAP states, Dynamic BC operation interface

**RESULTS**

**Table S1** Demographic Information for Case and Control Groups

| **Variable** | **Healthy**  **Controls (n=38)** | | **Schizophrenia Patients (n=42)** | | **Statistic**  **Analysis^a^** | |
| --- | --- | --- | --- | --- | --- | --- |
|  | M | SD | M | SD | *t* | *p* |
| Education | 11.05 | 2.91 | 10.48 | 2.84 | 0.90 | 0.373 |
| Age (years) | 24.76 | 4.56 | 24.86 | 4.80 | 0.09 | 0.929 |
|  | Yes | No | Yes | No | *χ*^2^ | *p* |
| Handedness (right) | 38 | 0 | 42 | 0 |  |  |
| Gender (male) | 25 | 13 | 27 | 15 | 0.02 | 0.888 |

^a^ two-sample t test

**Table S2** Longitudinal Alterations of Patients’ Clinical Symptoms

| **Variables** | **Patients before treatment (n=42)** | | **Patients after treatment (n=38)^a^** | | **Analysis ^b^** | |
| --- | --- | --- | --- | --- | --- | --- |
|  | M | SD | M | SD | *t* | *p* |
| PANSS-P | 25.60 | 3.75 | 15.39 | 2.98 | 19.15 | <0.001 |
| PANSS-N | 18.17 | 5.21 | 16.76 | 4.44 | 1.94 | 0.06 |

^a^ Four patients withdrew the follow-up measurements; ^b^ paired-sample t test. PANSS = Positive and Negative Syndrome Scale; PANSS-P = PANSS positive symptom scores; PANSS-N = PANSS negative symptom scores.

**Table S3** Abnormal Putamen-Related CAPs in Schizophrenia

| **Patients_0W *vs.* Controls** | **Differences in Brain Areas** | **Hemisphere** | **MNI Coordinates** | | | ***t*** |
| --- | --- | --- | --- | --- | --- | --- |
|  |  |  | **X** | **Y** | **Z** |  |
| ROI: putamen | thalamus | Left | -15 | -12 | 3 | 4.20 |
|  |  | Right | 15 | -12 | -3 | 4.27 |
|  | supplementary motor area | Left | -6 | 18 | 45 | 4.11 |
|  |  | Right | 6 | 18 | 45 | 4.41 |
|  | medial and paracingulate gyrus | Left | 9 | 18 | 33 | 4.08 |
|  |  | Right | -9 | 15 | 33 | 4.42 |
|  | paracentral lobule | Left | -12 | -27 | 69 | 3.16 |
|  | medial superior frontal gyrus | Left | 0 | 36 | 33 | 2.73 |
|  | anterior cingulate gyrus | Left | -6 | 21 | 27 | 3.19 |

Abbreviations: CAPs, coactivation patterns; ROI, regions of interest; MNI, Montreal Neurological Institute.

**Table S4**  Longitudinal Alterations of Pallidum- and Putamen- Related CAPs after Treatment

| **Patients_0W *vs* Patients_8W** | **Differences in Brain Areas** | **Hemisphere** | **MNI Coordinates** | | | ***t*** |
| --- | --- | --- | --- | --- | --- | --- |
|  |  |  | **X** | **Y** | **Z** |  |
| ROI: pallidum | Lingual gyrus | Left | -21 | -60 | -9 | 4.78 |
|  | fusiform | Left | -30 | -48 | -9 | 4.04 |
|  | middle temporal gyrus | Left | -63 | -54 | 12 | 4.24 |
|  | superior temporal gyrus | Left | -63 | -51 | 12 | 3.48 |
|  | angular gyrus | Left | -51 | -60 | 30 | 3.35 |
| ROI: putamen | lingual gyrus | Left | -21 | -96 | -18 | -3.34 |

Abbreviations: CAPs, coactivation patterns; ROI, regions of interest; MNI, Montreal Neurological Institute.
